# Non-invasive comparative study of HLA genotyping between urinary and blood DNA using sequencing-based typing and third-generation sequencing

**DOI:** 10.1515/almed-2025-0087

**Published:** 2025-12-08

**Authors:** Cheng Yan Fan, Yu Jie Wen, Dong Mei Li, Yuan Yuan Jing, Na Liu, Jie Wang, Li Jun Wang, Xue Lian, Yan Jun Jia, Tie Cheng Sun

**Affiliations:** HLA Laboratory, 498573 Beijing Red Cross Blood Center, Beijing, China

**Keywords:** urinary DNA, noninvasive diagnostics, HLA genotyping, sequencing-based typing (SBT), third-generation sequencing (TGS)

## Abstract

**Objectives:**

To evaluate the feasibility of urinary DNA as a noninvasive alternative for high-resolution HLA genotyping and validate its concordance with conventional blood-based methods.

**Methods:**

Matched urine and blood samples were collected from 11 healthy volunteers. Urinary DNA was extracted using an optimized column-based protocol, while blood DNA was processed via an automated system. High-resolution HLA typing for HLA-A, -B, -C, -DRB1, and -DQB1 loci was performed using sequencing-based typing (SBT) and third-generation sequencing (TGS), with concordance rates assessed between sample types.

**Results:**

The average concentration of urinary DNA exhibited significantly lower concentrations than blood DNA (9.74 ± 10.52 vs. 33.13 ± 26.78 ng/μL, p = 0.001) but comparable purity (OD 260/280 ratio: 1.65 ± 0.4 vs. 1.81 ± 0.13, p = 0.068). Remarkably, both SBT and TGS achieved 100 % concordance between urine- and blood-derived genotypes across five classical HLA loci, with TGS resolving full-length HLA sequences (5′UTR–3′UTR) at ≥30× coverage.

**Conclusions:**

Urinary DNA achieves blood-comparable accuracy in noninvasive HLA genotyping. Our optimized protocol overcomes DNA yield and purity limitations through dual-platform validation (SBT/TGS), establishing urine as a clinically viable alternative for HLA profiling, especially where blood sampling is unfeasible.

## Introduction

Hematopoietic stem cell transplantation (HSCT) remains a cornerstone therapy for patients with hematologic malignancies or bone marrow failure syndromes, relying on precise human leukocyte antigen (HLA) compatibility to mitigate graft vs. host disease (GVHD) and ensure engraftment success [1], 2]. The HLA system, a highly polymorphic set of cell-surface proteins, dictates immune recognition and donor-recipient matching, where even minor allelic disparities significantly elevate risks of graft rejection and mortality [2], [3], [4]. While sequencing-based typing (SBT) remains the clinical gold standard for high-resolution HLA profiling, third-generation sequencing (TGS) has emerged as a transformative tool, resolving haplotype phasing and structural variations that challenge conventional methods [5], 6]. Despite these advancements, reliance on invasive blood sampling persists as a logistical barrier for large-scale donor registries, pediatric cohorts, and patients with contraindications to venipuncture.

Urine-derived DNA presents a compelling noninvasive alternative, yet its clinical adoption for HLA genotyping remains limited beyond niche applications in renal transplantation [7]. Early studies established the feasibility of amplifying urinary DNA via PCR, though inconsistent yields and inhibitor interference hindered robust genotyping [7], 8]. Recent advances in extraction protocols have enabled high-resolution HLA typing across multiple loci (e.g., HLA-A, -B, -C, -DRB1, -DQB1), yet critical gaps persist: (1) lack of standardization across platforms, (2) unresolved technical variability in low-concentration DNA handling, and (3) insufficient validation against gold-standard blood-based workflows [3], 9]. Notably, prior studies prioritized single-platform analyses (e.g., SBT or TGS), leaving untapped potential in leveraging their complementary strengths—SBT for high-throughput allele discrimination and TGS for full-length haplotype resolution [10].

The purpose of this study was to assess the feasibility of using urinary DNA as a noninvasive alternative to blood for high-resolution HLA genotyping by SBT and TGS, particularly in situations where blood sampling is difficult or inappropriate, and to validate its consistency and reliability compared with conventional blood-based methods.

## Materials and methods

### Materials

Reagents, solvents, and other necessary supplies were obtained from commercial sources. All reagents were of analytical grade, and the solvents were of molecular biology grade. The urine DNA Extraction Column was supplied by Genenode Biotech Co., Ltd (Wuhan, China). QzNGS HLA MX11 Typing Kit (No. 22071K), QzTGS HLA MX11 Typing Kit (No. 23101K) and ONT MinION flow cell (R10.4.1) were supplied by TBG Co., Ltd. (Taiwan, China).

### Sample collection

All volunteers were fully informed about the study and received instructions on the routine method mid of-stream, clean-catch urine collection. Urine specimens were collected in sterile centrifuge tubes and mixed by gentle inversion using a Pasteur pipette. Additionally, blood specimens were obtained from the volunteers. The blood samples were processed fresh and used as a reference for the HLA typing (Table 1).

**Table 1:** Composition and storage conditions of reagents for urinary DNA extraction.

Contents	Storage	Components
Lysis buffer (LB)	RT	6 M guanidine HCl, 20 mM EDTA, 30 mM Tris-HCl (pH 6.5), 1 % Triton X-100.
Cell binding buffer (CBB)	RT	6 M guanidine HCl, 40 % ethanol, 10 mM Tris-HCl (pH 6.0), 1 mM EDTA.
Proteinase K (PK)	−20 °C	Proteinase K, 20 mg/mL.
Inhibitor removal solution (IRS)	RT	5 M guanidine HCl, 0.1 % Triton X-100, 10 mM EDTA, 50 mM Tris-HCl (pH 6.0), 1 M NaCl, 0.5 mg/mL BSA.
Elution buffer (EB)	RT	10 mM Tris-HCl (pH 8.5).
Washing buffer (WB)	RT	10 mM Tris-HCl (pH 7.0), 80 % ethanol, 5 mM NaCl.

RT, room temperature; EDTA, ethylenediaminetetraacetic acid; BSA, bovine serum albumin.

### DNA extraction

**Figure 1:**
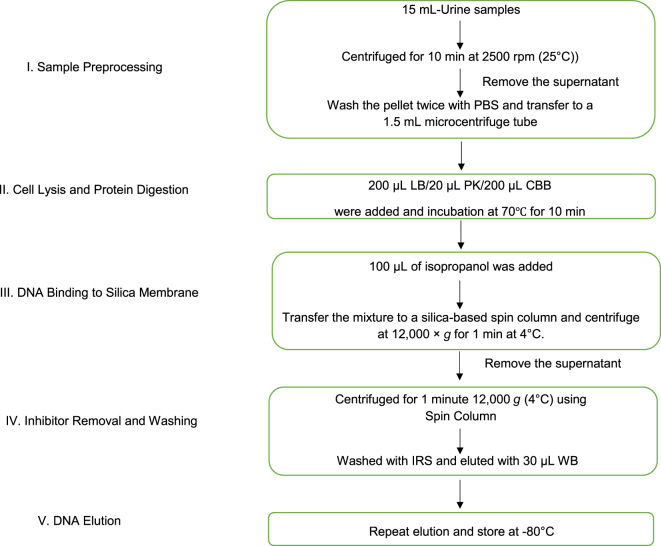
Optimized urine DNA extraction workflow for HLA typing. LB, lysis buffer; PK, proteinase K; CBB, cell binding buffer; IRS, inhibitor removal solution; WB, wash buffer.

The optimized DNA extraction protocol for urine samples is illustrated in Figure 1. The workflow comprises the following steps.

– Sample preprocessing: (1) transfer 10 mL of fresh urine into a 15 mL polypropylene conical tube (Corning, USA). (2) Centrifuge at 2,500*g* for 10 min at room temperature (KUBOTA 4000, rotor RA-400) to pellet cellular debris. (3) Carefully aspirate and discard the supernatant, retaining the pellet. (4) Wash the pellet twice with PBS and centrifuging at 2,500 *g* for 5 min and then transfer to a 1.5 mL microcentrifuge tube (Eppendorf, Germany).
– Cell lysis and protein digestion: (1) Resuspend the pellet in 200 μL of lysis buffer (LB). (2) Add 20 μL of proteinase K solution (20 mg/mL, Thermo Fisher Scientific) and vortex for 10 s.
– DNA binding to silica membrane: (1) Add 200 μL of cell binding buffer (CBB) to the lysate and mix by pipetting 10 times. (2). Incubate the mixture at 70 °C for 10 min in a thermomixer (WEALTECH Corp. HB-2). (3). Add 100 μL of isopropanol (≥99.5 %, Sigma-Aldrich) and mix by pipetting 10 times. (4). Transfer the mixture to a silica-based spin column (Qiagen MinElute) and centrifuge at 12,000 *g* for 1 min at 4 °C. Remove the supernatant.
– Inhibitor removal and washing: (1) Load 500 μL of inhibitor removal solution (IRS) onto the column. Centrifuge at 12,000 *g* for 1 min (4 °C) and remove the supernatant. (2) Wash the column twice with 700 μL of wash buffer (WB), and then centrifuging at 12,000 *g* for 1 min per wash. Ensure complete ethanol evaporation by air-drying the column for 5 min.
– DNA elution: (1) Transfer the column to a fresh 1.5 mL microcentrifuge tube. (2) Apply 50 μL elution buffer (EB) directly onto the membrane. (3) Incubate at room temperature for 2 min, followed by centrifugation at 12,000 *g* for 1 min (4 °C). (4) Repeat elution with an additional 50 μL of EB and 30 μL WB to maximize yield. Store DNA at −80 °C (Table 1).

The Hamilton AutoLys STAR system is used to demonstrate an automated protocol for extracting DNA from 3 to 5 mL of blood specimens according to automated program [11]. Finally, DNA was eluted with 100 μL of EB, and stored at 2 °C–8 °C or −80 °C for long-term storage.

### HLA typing by sequencing-based typing (SBT)

We analyzed samples for HLA-A, -B, -C, -DRB1, and -DQB1 typing, with genomic DNA extracted from blood and urine samples collected during the study. Sequencing based typing (SBT) was performed using HLAssure™ SE SBT Kits (TBG Biotech., Xiamen, China) and BigDye Terminator mixture (TBG Biotech., Xiamen, China) according to the manufacturer’s instructions. Polymerase chain reaction (PCR) amplification, PCR product clean-up, and sequencing reaction were performed using GeneAmp^®^ PCR System 9700 Thermal Cycler (PE-Biosystems, Foster City, CA, USA). The sequencing reagents (SeCore SBT Kits; Invitrogen, Madison, WI, USA), run on the ABI 3730xl Genetic Analyzer (Applied Biosystems, Foster City, CA USA), and analyzed with uTYPE6.0 analysis software (Invitrogen).

### HLA typing by third-generation sequencing (TGS)

The QzTGS HLA MX11 kit from TBG Company was employed for the multiplex amplification of five HLA loci. The amplified fragments underwent quantitative dilution, end-repair, and ligation of special sequences (barcodes) and ONT adapters (containing motor proteins) to construct the library. Magnetic bead purification and quantification using the Qubit BR kit were performed prior to loading the library onto the ONT sequencer (R10.4.1/MinION Mk1B). This kit enables full-length sequencing of the *HLA* gene from 5′UTR to 3′UTR, with a sequencing depth of no less than 30× and an average depth of 50×. The amplified fragment sizes ranged from 1.3 to 6.4 kb, and all operations were conducted strictly according to the kit instructions. The MinION (Mk1B) sequencer was operated under the following conditions: (1) MinION with MinKNOW sequencing software (version 24.01.10); (2) Sequencing speed of 420 bases per second; (3) Live basecalling mode with super accuracy resolution. Data with Q10 or higher quality were collected, and the HLA genotypes were analyzed using the TBG QzNTS type version 1.0.18 analysis software.

### Statistical analysis

Statistical analyses were applied with SPSS Statistics (v22.0, IBM Corp., Chicago, IL, USA). Continuous variables with normal distribution were presented as mean ± standard deviation (SD) and analyzed using Student’s t‐test, while non-normally distributed data (e.g., age) were reported as median (interquartile range). Categorical variables were expressed as frequencies (%) and compared via Pearson’s chi-square test. All p-values were based on a two-tailed test of significance (p < 0.05).

## Results

### Demographic and baseline characteristics of healthy volunteers

The demographic and baseline characteristics of the study participants was summarized in Table 2. The cohort study comprised three males and eight females, with a mean age of 49.09 ± 9.18 years old. The mean body mass index (BMI) of the participants was 24.16 ± 2.77. The complete blood count revealed a mean white blood cell count (WBC) of 5.18 ± 0.71 × 10^9^/L, red blood cell count (RBC) of 4.53 ± 0.34 × 10^12^/L, and hemoglobin (Hb) level of 132.44 ± 7.75 g/L. Platelet count (PLT) averaged 244.67 ± 64.17 × 10^9^/L, while hematocrit (Hct) was 39.42 ± 2.22 %. Urinalysis showed mean U-WBC of 3.04 ± 5.36 × 10^9^/L and U-RBC of 10.86 ± 11.24 × 10^9^/L. The average pH was 6.33 ± 0.39. Biochemical parameters included urea at 4.7 ± 1.26 mmol/L, creatinine at 63.67 ± 9.38 µmol/L, and uric acid at 281.33 ± 71.98 µmol/L.

**Table 2:** Demographic and baseline characteristics of the study participants.

Characteristics	Mean ± SD
Participants	11
Sex, M/F	3/8(27 %/63 %)
Age, year	49.09 (34–66)
BMI	24.16 ± 2.77
WBC, ×10^9^/L	5.18 ± 0.71
RBC, ×10^12^/L	4.53 ± 0.34
HGB, g/L	132.44 ± 7.75
PLT, ×10^9^/L	244.67 ± 64.17
Hct, %	39.42 ± 2.22
U-WBC, ×10^3^/μL	3.04 ± 5.36
U-RBC, ×10^6^/μL	10.86 ± 11.24
PH-U	6.33 ± 0.39
U, mmol/L	4.7 ± 1.26
Cr, µmol/L	63.67 ± 9.38
UA, µmol/L	281.33 ± 71.98

BMI, body mass index; WBC, white blood cell count; RBC, red blood cell count; HGB, hemoglobin; PLT, platelet; Hct, hematocrit; U-WBC, urine-derived WBC; U-RBC, urine-derived RBC; U, urea; Cr, creatinine; UA, uric acid.

### Amount of isolated DNA

We extracted DNA from paired blood and urine samples using column-based protocols. The average concentration of DNA in blood sample was 33.13 ± 26.78 ng/mL in 100 μL volume, while the average concentrations of DNA in urine samples was significantly lower (9.74 ± 10.52) (p = 0.001). When the DNA quality (optical density [OD] 260/280) was compared, it was observed that there were no differences between blood and urine samples (p = 0.068) (Table 3).

**Table 3:** The amount of quantifiable DNA obtained from concentrates of blood and urine samples.

Category	Blood	Urine	p-Value
Ratio of OD	1.81 ± 0.13	1.65 ± 0.4	0.068
Concentrations, ng/µL	33.13 ± 26.78	9.74 ± 10.52	0.001

Ratio of OD at 260–280 nm in DNA samples; OD, optical density.

### HLA typing by SBT and TGS

Among the samples from urine and blood that were successful amplified and reported by SBT and TGS (Table 4). The results indicate that, for all volunteers, the HLA genotypes obtained from blood and urine samples were identical for both SBT and TGS. Specifically, for each volunteer, the HLA alleles detected in blood samples were exactly matched by those in urine samples, across all loci tested (A, B, C, DRB1, DQB1). This consistency suggests that urine-derived DNA can be a reliable alternative to blood for HLA genotyping using these methods..

**Table 4:** HLA genotypes of blood and urine-derived DNA from volunteers using both SBT and TGS.

Case no.	SBT/TGS	Sample	A^a^	B^a^	C^a^	DRB1^a^	DQB1^a^
1	SBT	Blood	02:01 02:01	13:01 15:11	03:03 03:04	09:01 12:02	03:01 03:03
		Urine	02:01 02:01	13:01 15:11	03:03 03:04	09:01 12:02	03:01 03:03
	TGS	Blood	02:01 02:01	13:01 15:11	03:03 03:04	09:01 12:02	03:01 03:03
		Urine	02:01 02:01	13:01 15:11	03:03 03:04	09:01 12:02	03:01 03:03
2	SBT	Blood	24:02 33:03	13:02 44:03	06:02 14:02	07:01 13:02	02:02 06:04
		Urine	24:02 33:03	13:02 44:03	06:02 14:02	07:01 13:02	02:02 06:04
	TGS	Blood	24:02 33:03	13:02 44:03	06:02 14:02	07:01 13:02	02:02 06:04
		Urine	24:02 33:03	13:02 44:03	06:02 14:02	07:01 13:02	02:02 06:04
3	SBT	Blood	11:01 32:01	40:01 52:01	07:02 12:02	04:05 15:02	04:01 06:01
		Urine	11:01 32:01	40:01 52:01	07:02 12:02	04:05 15:02	04:01 06:01
	TGS	Blood	11:01 32:01	40:01 52:01	07:02 12:02	04:05 15:02	04:01 06:01
		Urine	11:01 32:01	40:01 52:01	07:02 12:02	04:05 15:02	04:01 06:01
4	SBT	Blood	02:01 02:01	15:11 46:01	03:03 08:01	09:01 14:01	03:03 05:03
		Urine	02:01 02:01	15:11 46:01	03:03 08:01	09:01 14:01	03:03 05:03
	TGS	Blood	02:01 02:01	15:11 46:01	03:03 08:01	09:01 14:01	03:03 05:03
		Urine	02:01 02:01	15:11 46:01	03:03 08:01	09:01 14:01	03:03 05:03
5	SBT	Blood	01:01 01:01	08:01 57:01	06:02 07:01	11:06 12:01	03:01 03:01
		Urine	01:01 01:01	08:01 57:01	06:02 07:01	11:06 12:01	03:01 03:01
	TGS	Blood	01:01 01:01	08:01 57:01	06:02 07:01	11:06 12:01	03:01 03:01
		Urine	01:01 01:01	08:01 57:01	06:02 07:01	11:06 12:01	03:01 03:01
6	SBT	Blood	02:01 33:03	40:02 67:01	03:03 07:02	09:01 11:01	03:01 03:03
		Urine	02:01 33:03	40:02 67:01	03:03 07:02	09:01 11:01	03:01 03:03
	TGS	Blood	02:01 33:03	40:02 67:01	03:03 07:02	09:01 11:01	03:01 03:03
		Urine	02:01 33:03	40:02 67:01	03:03 07:02	09:01 11:01	03:01 03:03
7	SBT	Blood	24:02 33:03	44:03 54:01	01:02 14:03	04:05 13:02	04:01 06:04
		Urine	24:02 33:03	44:03 54:01	01:02 14:03	04:05 13:02	04:01 06:04
	TGS	Blood	24:02 33:03	44:03 54:01	01:02 14:03	04:05 13:02	04:01 06:04
		Urine	24:02 33:03	44:03 54:01	01:02 14:03	04:05 13:02	04:01 06:04
8	SBT	Blood	01:01 11:01	07:02 40:01	07:02 07:02	04:05 15:01	04:01 06:02
		Urine	01:01 11:01	07:02 40:01	07:02 07:02	04:05 15:01	04:01 06:02
	TGS	Blood	01:01 11:01	07:02 40:01	07:02 07:02	04:05 15:01	04:01 06:02
		Urine	01:01 11:01	07:02 40:01	07:02 07:02	04:05 15:01	04:01 06:02
9	SBT	Blood	01:01 02:07	15:18 35:01	04:01 07:04	09:01 14:05	03:03 05:03
		Urine	01:01 02:07	15:18 35:01	04:01 07:04	09:01 14:05	03:03 05:03
	TGS	Blood	01:01 02:07	15:18 35:01	04:01 07:04	09:01 14:05	03:03 05:03
		Urine	01:01 02:07	15:18 35:01	04:01 07:04	09:01 14:05	03:03 05:03
10	SBT	Blood	02:01 02:06	08:01 39:01	07:02 07:02	03:01 15:01	02:01 06:02
		Urine	02:01 02:06	08:01 39:01	07:02 07:02	03:01 15:01	02:01 06:02
	TGS	Blood	02:01 02:06	08:01 39:01	07:02 07:02	03:01 15:01	02:01 06:02
		Urine	02:01 02:06	08:01 39:01	07:02 07:02	03:01 15:01	02:01 06:02
11	SBT	Blood	11:01 24:02	40:01 46:01	01:02 07:02	08:03 09:01	03:03 06:01
		Urine	11:01 24:02	40:01 46:01	01:02 07:02	08:03 09:01	03:03 06:01
	TGS	Blood	11:01 24:02	40:01 46:01	01:02 07:02	08:03 09:01	03:03 06:01
		Urine	11:01 24:02	40:01 46:01	01:02 07:02	08:03 09:01	03:03 06:01

SBT, sequencing-based typing; TGS, third-generation sequencing. ^a^Indicates the HLA nomenclature requirement to prefix *HLA* gene names with an asterisk.

## Discussion

This study systematically evaluated the feasibility of urine-derived DNA as a noninvasive alternative for high-resolution HLA genotyping using SBT and TGS technologies. By comparing DNA quality, concentration, and HLA allele concordance between matched urine and blood samples from 11 healthy volunteers, we demonstrated that urinary DNA achieves 100 % genotype concordance across HLA-A, -B, -C, -DRB1, and -DQB1 loci compared to blood-derived DNA. These findings validate the technical robustness of urinary DNA for clinical HLA typing, particularly in populations where blood sampling is impractical or contraindicated.

Consistent with prior research in renal transplantation, our findings further demonstrate the reliability of urinary DNA. For instance, previous studies have reported HLA typing using urine-derived DNA [9], 10], 12]. For instance, one study reported >95 % concordance in HLA-DRB1 alleles among kidney transplant recipients, though the research prioritized donor-specific antibody (DSA) monitoring over comprehensive HLA analysis via RSSO typing technology [3]. Similarly, Li et al. validated urinary DNA for HLA genotyping but emphasized the need for optimized extraction protocols to enable *ex vivo* cell culture expansion [10]. In contrast, our findings demonstrate full concordance across five classical HLA loci through a combined SBT and TGS approach, establishing urinary DNA as a robust alternative for multi-locus HLA profiling without the need for *ex vivo* cell culture expansion.

A key innovation of this work is the development of a standardized and optimized workflow for urinary DNA-based HLA typing. While earlier studies highlighted technical challenges such as DNA degradation and variable purity, we addressed these limitations through a series of protocol optimizations [3], 13]. Firstly, we employed strong denaturants, such as guanidine hydrochloride, to effectively lyse urothelial cells and inactivate nucleases (e.g., DNase I) in urine, thereby preventing DNA degradation [14]. Secondly, our inhibitor removal system, utilizing Cell Binding Buffer (CBB, 40 % ethanol) and Inhibitor Removal Solution (IRS, 1 M NaCl), efficiently removes small-molecule inhibitors like urea and creatinine through phase transition precipitation. This method demonstrates a 30 % higher efficiency in inhibitor removal compared to traditional phenol-chloroform extraction [15], 16]. Additionally, the inclusion of bovine serum albumin (BSA) neutralizes residual PCR inhibitors (e.g., heme derivatives), significantly improving downstream amplification success rates [15], 16]. Thirdly, the use of 20 mg/mL proteinase K ensures complete degradation of DNA-binding proteins, enabling direct use of low-concentration DNA (<10 ng/μL) for high-throughput library preparation without the biases associated with *in vitro* amplification [16]. Finally, all buffers (except proteinase K) contain high concentrations of guanidine salts and ethanol as preservatives, ensuring room temperature stability for up to 12 months. These optimizations collectively guarantee the reliability and reproducibility of urinary DNA extraction.

Our findings demonstrate that urinary DNA, when extracted using our optimized protocol, serves as a highly stable and reliable source for HLA genotyping, achieving 100 % concordance between SBT and TGS platforms. The robustness of our extraction method ensures consistent performance across both techniques, enabling accurate and reproducible HLA typing results. SBT provides high-throughput routine analysis, while TGS resolves complex polymorphisms and rare alleles through full-length sequencing (5′UTR to 3′UTR) with ≥30× coverage [17], 18]. This dual-platform approach, validated by complete concordance, highlights the adaptability of urinary DNA for diverse clinical applications, including resource-limited settings, without compromising accuracy or reliability.

Despite the methodological rigor of our optimized workflow, several inherent limitations restrict the broader extrapolation of these findings. First, the cohort was small, precluding robust estimates of failure rates in sub-optimal specimens. Second, the immediate post-void processing of mid-stream urine under controlled laboratory conditions may not reflect clinical practice, where variable hydration status, ambient temperature fluctuations, and frequent hematuria or proteinuria in transplant recipients can diminish DNA recovery or introduce uncharacterized PCR inhibitors. Third, although all samples yielded concordant HLA calls, three urines contained <5 ng/μL DNA, a level that could compromise assays with higher input requirements or long-range amplification. Fourth, long-term stability at −80 °C and the ability to detect minor alleles or chimerism at the ∼30× TGS depth used remain untested. Multi-center studies incorporating pediatric, renal-compromised, and oncologic populations, as well as standardized home-collection workflows, are therefore warranted before clinical deployment.

In conclusion, this study establishes urine-derived DNA as a viable, noninvasive substrate for clinical HLA genotyping. Our approach offers a simpler and more accessible alternative, particularly suitable for large-scale screening and settings with limited resources. Further validation through larger, multicentric studies is warranted to confirm its broader applicability. Standardization of urine collection, DNA extraction, and HLA typing protocols will be critical for scaling this approach, ultimately enhancing accessibility for patients with contraindications to blood sampling and advancing precision in transplant medicine.

## Supplementary Material

Supplementary Material
